# The potential role of hybridization in diversification and speciation in an insular plant lineage: insights from synthetic interspecific hybrids

**DOI:** 10.1093/aobpla/plx043

**Published:** 2017-09-01

**Authors:** Benjamin Kerbs, Jacob Ressler, John K Kelly, Mark E Mort, Arnoldo Santos-Guerra, Matthew J S Gibson, Juli Caujapé-Castells, Daniel J Crawford

**Affiliations:** 1 Department of Biological Sciences, Emporia State University, Emporia, KS 66801, USA; 2 Department of Biology, Indiana University, Bloomington, IN 47405, USA; 3 Department of Ecology & Evolutionary Biology, University of Kansas, Lawrence, KS 66045-7534, USA; 4 Calle Guaidil 16, Urbanización Tamarco, 38280 Tegueste, Tenerife, Canary Islands, Spain; 5 Department of Biology, Indiana University, Bloomington, IN 47405, USA; 6 Jardín Botánico 13 Canario “Viera y Clavijo”-Unidad Asociada al CSIC (Cabildo de Gran Canaria), Camino del palmeral 14 15 (Tafira Alta), 35017 Las Palmas de Gran Canaria, Spain; 7 Department of Ecology & Evolutionary Biology, and the Biodiversity Institute, University of Kansas, Lawrence, KS 66045-7534, USA

**Keywords:** Canary Islands, phenotypic groups, synthetic hybrids, transgressive traits

## Abstract

Hybridization is recognized as an important process in plant evolution, and this may be particularly true for island plants where several biotic and abiotic factors facilitate interspecific hybridization. Although rarely done, experimental studies could provide insights into the potential of natural hybridization to generate diversity when species come into contact in the dynamic island setting. The potential of hybridization to generate morphological variation was analysed within and among 12 families (inbred lines) of an F_4_ hybrid generation between two species of *Tolpis* endemic to the Canary Islands. Combinations of characters not seen in the parents were present in hybrids. Several floral and vegetative characters were transgressive relative to their parents. Morphometric studies of floral, vegetative and fruit characters revealed that several F_4_ families were phenotypically distinct from other families, and from their parents. The study demonstrates that morphologically distinct pollen-fertile lines, potentially worthy of taxonomic recognition if occurring in nature, can be generated in four generations. The ability of the hybrid lines to set self-seed would reduce gene flow among the lines, and among the hybrids and their parental species. Selfing would also facilitate the fixation of characters within each of the lines. Overall, the results show the considerable potential of hybridization for generating diversity and distinct phenotypes in island lineages.

## Teaser

Islands contain some of the most remarkable yet imperiled plants anywhere on Earth. Despite remarkable ecological/morphological diversity, insular endemics often retain the ability to hybridize and produce vigorous, fertile progeny. This study over several generations of synthetic interspecific hybrids between two species endemic to the Canary Islands shows that gene exchange between species can produce novel traits, transgressive phenotypes and novel combinations of features not seen in their parents. Results of this study provide experimental evidence of the potential of hybridization to promote diversification and possibly generate stabilized hybrid derivatives in plants in an oceanic archipelago.

## Introduction

The prevalence and significance of hybridization in evolution have been debated over the past decades with zoologists tending to minimize its impact (e.g. Mayr 1942; Dobzhansky 1970) and botanists, with rare exceptions (Wagner 1970), seeing a more important role for hybridization (Anderson and Stebbins 1954; Stebbins 1959; Raven 1976; Arnold 2016). However, it is now widely accepted that hybridization is an important factor in plant evolution and indeed in the evolution of many other groups of organisms (Arnold 2015, 2016; Mallet *et al.* 2016). Hybrids may display traits exceeding those found in their parents, i.e. transgressive traits (Rieseberg et al. 1999, 2003b; Stelkens and Seehausen 2009; Yakimowski and Rieseberg 2014), as well as exhibit novel combinations of traits from the two parents (Rieseberg and Ellstrand 1993; García-Verdugo *et al.* 2013). Both transgressive traits and new character combinations can facilitate evolutionary change in plant lineages (Stebbins 1959; Rieseberg *et al.* 2003a; Arnold *et al.* 2012; Arnold 2016), including the establishment and evolution of independent homoploid lineages that may be recognized as species (Abbott *et al.* 2010; Schumer *et al.* 2014; Yakimowski and Rieseberg 2014). Speciation associated with hybridization and increase in ploidy level is especially prevalent in flowering plants (Soltis *et al.* 2016).

Several factors make natural interspecific hybridization feasible in plant lineages on oceanic islands, most notably the combination of few intrinsic reproductive barriers with a dynamic ecological landscape. Species are typically isolated by ecological and spatial factors rather than intrinsic barriers (Crawford and Stuessy 1997; Crawford and Archibald 2016). The lack of intrinsic barriers has been confirmed in multiple lineages where fertile interspecific hybrids have been synthesized (Gillett and Lim 1970; Lowrey 1986; Mayer 1991; Brochmann *et al.* 2000; Carr 2003). Congeneric species often occupy distinct habitats but produce vigorous, fertile hybrids when they come into contact (e.g. Francisco-Ortega *et al.* 1997; Brochmann *et al.* 2000; Carr 2003). The insular landscape is dynamic, where natural and anthropogenic disturbances can bring species into contact and provide potential habitats for hybrids throughout different stages of island ontogeny. For example, It has been estimated that 10 % of the Hawaiian flora has been involved in natural hybridization (Whitney *et al.* 2010). Anderson and Stebbins (1954), in a classic paper on the impacts of hybridization on evolution, highlighted oceanic islands as places with changing environmental conditions that facilitate rapid bursts of hybridization.

In the present study, the genus *Tolpis* (Asteraceae) was used to examine the diversity generated by interspecific hybridization. This is a small (10–15 species) monophyletic group occurring in the Mediterranean and North Africa with its centre of diversity in the Canary Islands (Jarvis 1980; Gruenstaeudl *et al.* 2013; Mort *et al.* 2015). Despite being a small radiation Canarian *Tolpis* have breeding systems that range from self-incompatible (SI), through pseudo-self-compatible (PSC), to fully self-compatible (SC; Crawford et al. 2008, 2015). There is also variation in habit (perennials and an annual), floral and vegetative morphology, ploidy level and habitat preference (Jarvis 1980; Crawford *et al.* 2008). First generation hybrids have been obtained between Canary Island *Tolpis*, with reduced pollen fertility in some hybrids (Jarvis 1980; Crawford et al. 2009, 2015). In addition to the variation, Canarian *Tolpis* is an ideal subject for experimental hybridization because plants are easily cultivated in large numbers, are easily manipulated and have relatively short generation times, typically about 2 months.

**Figure 1.**
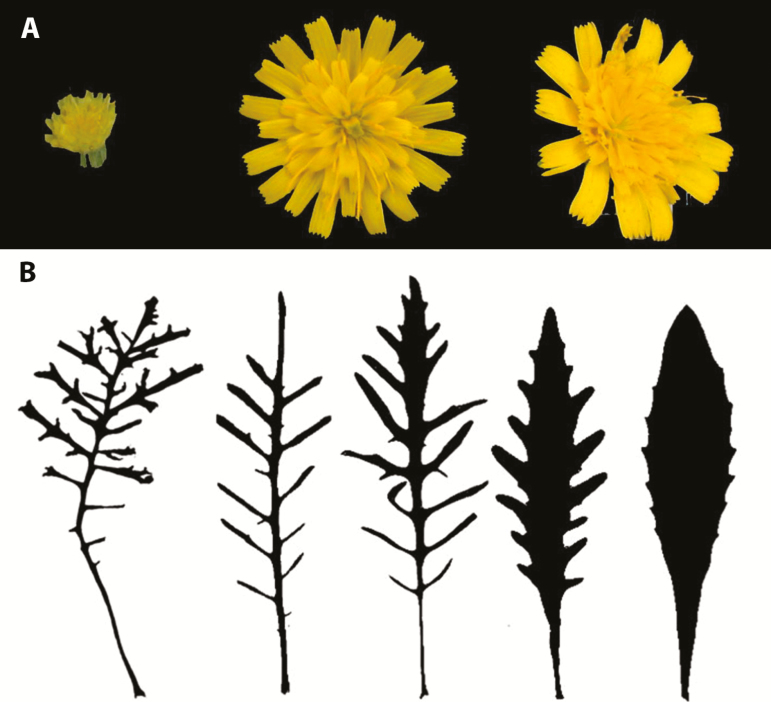
(A) Capitula of *Tolpis coronopifolia* (left), *T. santosii* (right) and an F_1_ hybrid (middle). (B) Leaf silhouettes of *T. coronopifolia* (left), *T. santosii* (right) and representative F_4_ hybrids (middle three). Photo J. K. Archibald.

We produced inbred lines starting with a cross between an annual, SC species and a perennial SI/PSC species. The SC annual *Tolpis coronopifolia*. It is the only Canarian species with the ‘selfing syndrome’ (Ornduff 1969; Slotte *et al.* 2012) including fewer florets per capitulum and smaller florets compared to outcrossing members of the genus (Fig. 1A). The species has dissected leaves (Fig. 1B) and occurs only on Tenerife Island where it grows in open habitats from 150 to 1400 m above sea level. The other parental species is the recently described *T. santosii* (Crawford *et al.* 2013), which is a SI to PSC perennial with large capitula (Fig. 1A). This species was chosen because it is a strong perennial that persists for a decade or longer in the greenhouse, and it grows and flowers profusely under cultivation. In contrast to *T. coronopifolia*, the leaves of *T. santosii* are nearly entire (Fig. 1B). The species is distributed only along the north and northeast coast of the island of La Palma.

Carr (2003) suggested that the ability to hybridize over time is more important in an evolutionary context than the hybrids present at any one time because the natural hybrids occurring at any given time will depend on the distribution of the parents. This means that the past and future roles of hybridization may be grossly underestimated if based only on the occurrence of natural hybrids, and that the results of artificial hybridization must be incorporated into estimates of the potential evolutionary significance of hybridization within a lineage. In oceanic islands, many factors may change the distributions of species over time. For example, human activities have resulted in the purposeful movement of endemic species among islands for use as ornamentals in gardens and roadsides (Francisco-Ortega *et al.* 2000), creating the potential for hybridization among species with weak post-zygotic isolating barriers. In addition, disturbances from human activities, such as road construction, facilitate the formation and establishment of interspecific hybrids when formerly isolated species come into contact (Brochmann *et al.* 2000). Over a larger temporal scale, Brennan *et al.* (2014) discuss climate change and hybridization, and Vallejo-Marín and Hiscock (2016) argue that species isolated by pre-zygotic barriers will likely be more affected by climate change than those with strong intrinsic post-zygotic barriers. As indicated earlier, most island plants, including *Tolpis*, fall into the former category. The parental species of *Tolpis* used in the present study occur on different islands and are not known to hybridize in nature. However, one or more of the factors mentioned above could affect species distribution in the future, especially human activities. While interspecific hybridization is apparently rare in Canarian *Tolpis*, examples are known. One example involves hybridization on Tenerife between sympatric populations of *T*. *coronopifolia* and the perennial *T. webbii* (J. K. Archibald and D. J. Crawford, University of Kansas, unpubl. data). These considerations suggest that results of the current study can provide some appreciation of the potential of hybridization to generate novelty and diversity when various factors bring species of *Tolpis* come into contact in the Canary Islands.

The major purpose of this study was to provide direct experimental evidence of the phenotypic diversity generated from hybridization between two species endemic to an oceanic archipelago. More specifically, we used inbred hybrid lines in *Tolpis* to: (1) determine whether any of the hybrid inbred lines form fertile, distinct phenotypic groups and (2) ascertain whether there are transgressive traits or novel combinations of traits were generated by in the hybridization.

## Methods

**Figure 2.**
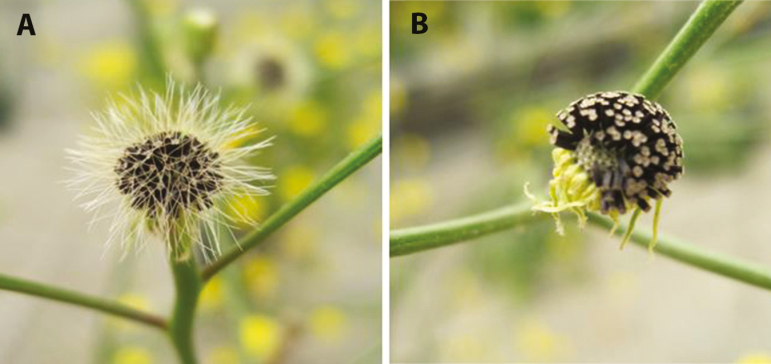
Fruits with (A) and lacking (B) a pappus of setaceous hairs.

One plant of *T. coronopifolia* (Crawford *et al.* 2008) was used as the pollen parent in a cross with an individual of *T. santosii* (Crawford *et al.* 2013); all hybrids were grown in the greenhouses at the University of Kansas. Vouchers of the parental species used to generate F_4_ progeny are deposited in KANU under accession numbers: *Tolpis santosii* 397175 and *T. coronopifolia* 397158. High-quality digital images of the leaves of F_4_ progeny are available upon request. Seed from one self-pollinated F_1_ hybrid plant produced an F_2_ generation (Soto-Trejo *et al.* 2013) from which one plant with fruit lacking the typical pappus of setaceous hairs (Fig. 2B) was selfed to produce the F_3_ generation. Twelve F_3_ plants that represented much of the phenotypic variation seen in that generation were selfed to produce the F_4_ families for study. The F_3_ and F_4_ generations allow the hybrid heterozygosity to segregate out into the three genotypic classes. Mendelian segregation predicts that on average half of the loci heterozygous in the inter-species hybrid (F_1_) plant will be heterozygous in any one F_2_ plant, while F_4_ plants should be heterozygous at only about 12.5 % (1/8) of the loci that were heterozygous in the original F_1_. Whether a particular F_4_ plant is homozygous for the *T*. *coronopifolia* or *T. santosii* allele will vary, but should be positively correlated within F_4_ families (they will be identical at all loci that were homozygous in their particular F_3_ parent). Nearly all loci that were heterozygous in the founding F_2_ plant should be polymorphic in the F_4_ population (as a whole) with a predicted segregation ratio of 3:2:3 ratio (AA:AB:BB). A total of 188 F_4_ plants were established from 12 families, with the number of individuals limited primarily by the number of viable fruits produced in the F_3_ generation. Voucher specimens are deposited in the McGregor Herbarium (KANU) of the University of Kansas.

Morphometric methods, the quantitative analysis of quantitative and qualitative variables (Henderson 2006), were used to assess variation in the 12 F_4_ generation lines. A total of 15 characters (4 vegetative, 6 floral, 4 fruit and 1 pollen) were measured across individuals from each inbred line (Table 1). In addition, a leaf dissection index was calculated using the method of Kincaid and Schneider (1983). These inbred lines contained 5 to 25 individuals (mean 15.4), and 3 to 5 leaves were measured per individual. Pollen viability and self-seed set were measured as percentages. The seed (technically the fruit) mass (µg) was the sum value of 20 achenes.

**Table 1.** Characters measured in phenotypic study of *Tolpis* hybrids. Units in mm unless otherwise stated in parentheses.

Leaf	Floral	Fruit	Other
Leaf area	Capitulum diameter (cm)	Fruit mass (mg)	Pollen viability (%)
Leaf perimeter	Ligule length	Fruit length	
Leaf length	Ligule width	Fruit width	
Leaf width	Involucral bract width	Seed set (%)	
	Involucral bract length		
	Style branch length		

Per cent pollen fertility was determined for all plants by staining a minimum of 200 grains in lactophenol aniline blue (Kearns and Inouye 1993). The relatively large, darkly stained pollen grains were easily distinguished from the shrivelled, very lightly stained grains. The percentage of self-seed set $\left(\frac{number\;of\;seeds}{total\;number\;of\;florets}\right)$ was estimated for each plant. The large, plump, dark (dark brown to black) fruits contain embryos and are easily distinguishable from the light tan, shrunken fruits lacking embryos. The presence/absence of a pappus was scored for each of the inbred lines.

Vegetative characters were measured using herbarium digitizing techniques. A Canon 5D Mark III (Melville, NY, USA) and a Photo-eBox lighting system were used to image 350 pressed and dried leaves from F_4_ plants. Leaf measurements were taken using ImageJ (Schneider *et al.* 2012). Leaf length was measured from the base of the petiole to the apex of the leaf, and width was measured as the widest distance between lobes, typically half way up the midrib of the leaf. Leaf area and perimeter were calculated by increasing the image threshold and examining the number and boundaries of vegetative particles in the image. More specifically, an image of a leaf was taken with a ruler in the photo. The measurement feature was calibrated in ImageJ using the ruler, which allowed us to later make simple measurements of length and width, and also more complex measurements such as area and perimeter. The colour photo was converted to an 8-bit (greyscale) image and the threshold of the photo is increased so that vegetative particles are fully saturated/black against a white background (as in Fig. 1B). Since ImageJ was calibrated using the ruler, the program is able to analyse the pixels that represent the leaf and measures the leaf area. Similarly ImageJ can assign an outline to the cluster of particles/pixels and measure leaf perimeter. Instructions for these procedures are available online.

Following measurements of the diameter of 3 to 5 intact capitula per individual, the capitula were dissected and characters (ligule length and width, style branch length; bract length and width) were measured for 5 florets per capitulum. Structures were imaged using a digital Nikon dissecting microscope (×10) and measured using InfinityAnalyze (Lumenera, Ottawa, ON, Canada), which allows for simple length and width measurements. The software InfinityAnalyze produces a live image of the specimen under the lens of a digital microscope. After calibrating the measurement feature of the software using a ruler under the lens, it is possible to make length and width measurements on screen. These measurements were taken using software because the small and sometimes non-linear nature of the floral parts (e.g. curled style branches) precludes unmagnified measurement with a ruler. All seed data except per cent self-seed set and seed weight were collected using these techniques as well.

Means and standard errors were determined for each floral, vegetative and fruit character. Tukey–Kramer Honestly Significant Difference (HSD) *post hoc* tests and ANOVA were performed on each character singly to examine variance among the F_4_ inbred lines. Varying criteria have been employed for scoring hybrid traits as transgressive (Rieseberg *et al.* 1999; Stelkens and Seehausen 2009); in the present study, traits were considered transgressive if the mean values were outside the ranges of the two parents (Stelkens and Seehausen 2009).

Floral and vegetative data were pooled for analyses in multivariate space. Principal component analysis (PCA) was performed on the floral and vegetative characters and an individual factor map was created. Missing data were estimated using a non-parametric multiple imputation approach via the missMDA package (Dray and Josse 2015; Josse and Husson 2016) for R and the final data set was plotted using the associated FactomineR package. Ninety-five per cent barycentric confidence ellipses were assigned around group centroids.

## Results

**Figure 3.**
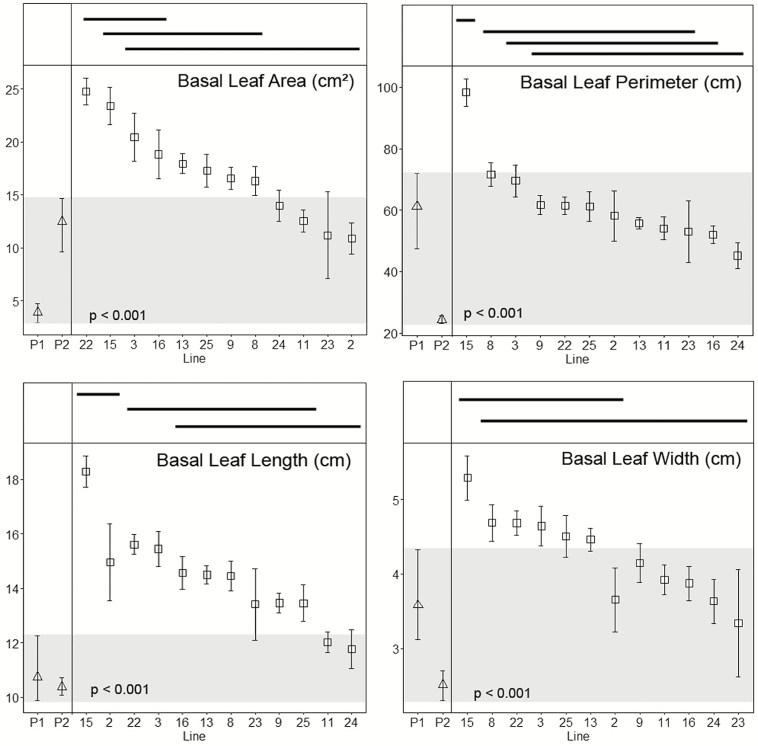
Plots of mean values (squares) and standard errors of inbred hybrid lines for vegetative characters. An ANOVA yields *P*-values < 0.05 for all characters, suggesting differences among lines. Bars above the graphs indicate which families are not significantly different from one another (*P* > 0.05, Tukey–Kramer HSD *post hoc* tests). Mean values (triangles) and ranges for the original progenitors, *Tolpis coronopifolia* (P1) and *T. santosii* (P2) are shown in the left-hand side of each plot. The greyed regions depict the ranges for the two parents. Hybrid line means falling outside this range represent transgressive traits. Lines are sorted mostly by decreasing mean values except lines 2 and 23 which exhibit large variability for some characters.

**Figure 4.**
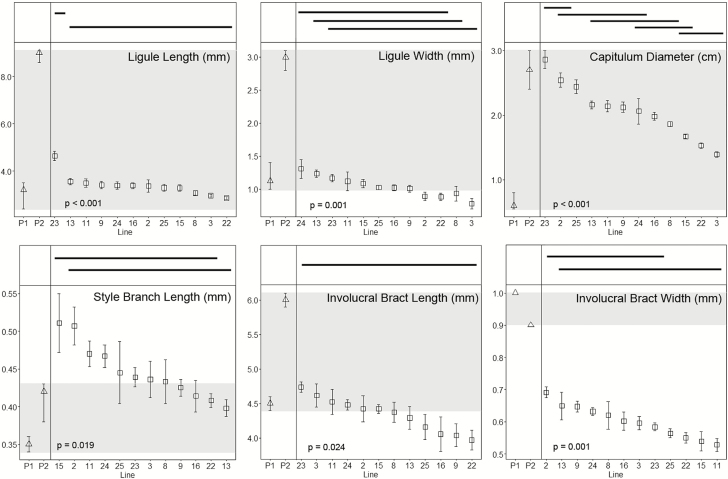
Plots of mean values (squares) and standard errors of inbred hybrid lines for floral characters. An ANOVA yields *P*-values < 0.05 for all characters, suggesting differences among lines. Bars above the graphs indicate which families are not significantly different from one another (*P* > 0.05, Tukey–Kramer HSD *post hoc* tests). Mean values (triangles) and ranges for the original progenitors, *Tolpis coronopifolia* (P1) and *T. santosii* (P2) are shown in the left-hand side of each plot. The greyed regions depict the ranges for the two parents. Hybrid line means falling outside this range represent transgressive traits. Involucral bract length was the only character that did not delimit lines into more than one group. Lines are sorted by decreasing mean values.

**Figure 5.**
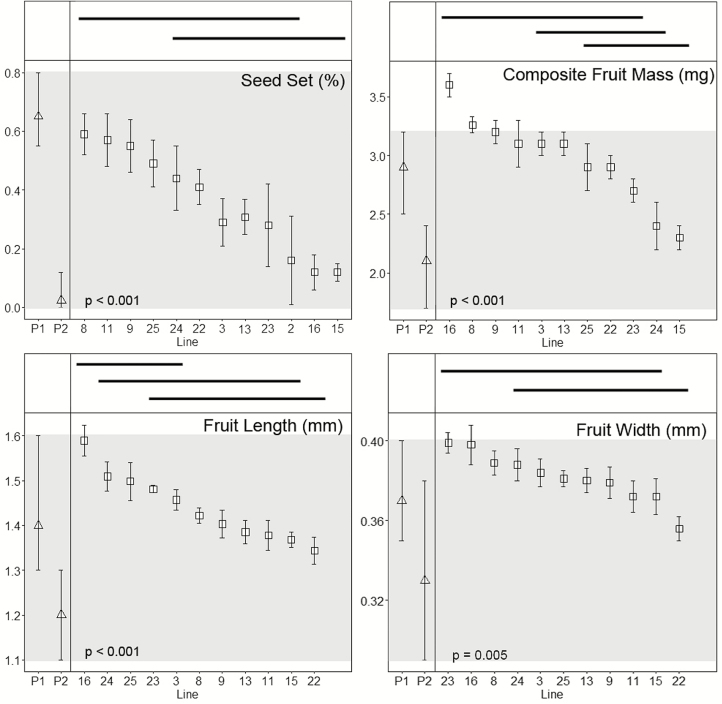
Plots of mean values (squares) and standard errors of inbred hybrid lines for fruit characters. An ANOVA yields *P*-values of < 0.05 for all characters, suggesting differences among lines. Bars above the graphs indicate which families are not significantly different from one another (*P* > 0.05, Tukey–Kramer HSD *post hoc* tests). Mean values (triangles) and ranges for the original progenitors, *Tolpis coronopifolia* (P1) and *T. santosii* (P2) are shown in the left-hand side of each plot. The greyed regions depict the ranges for the two parents. Hybrid line means falling outside this range represent transgressive traits. Inbred lines are sorted by decreasing mean values. Line 2 had an insufficient population size for fruit characters and was not included in the size analyses. Composite fruit mass is a measurement of 20 seeds from each inbred line.

All hybrids were perennials, with none of the plants flowering once and senescing, as is typical of the SC annual parent *T*. *coronopifolia*. Means and standard errors for floral, vegetative and fruit characters are shown in Figs 3–5, together with the means and ranges of the traits of their parents. For hybrids, each ANOVA test yielded *P*-values of <0.05, suggesting that each character examined varies significantly among inbred lines. Results of the Tukey–Kramer HSD *post hoc* tests are shown by the horizontal bars above the plots of means and standard errors shown in Figs 3–5. Involucral bract length is the only character that failed to delimit inbred lines into two or more groups (Fig. 4).

Line 15 was notable in that all leaf traits were transgressive to the parents, including being the only line that was transgressive for leaf perimeter (Fig. 3). By contrast, lines 11 and 24 were the only lines that were intermediate for all leaf traits. Two traits, basal leaf area and length, were transgressive in the majority of lines, whereas leaf perimeter was, with the exception of line 15, intermediate between the parents (Fig. 3). With regard to floral traits, neither ligule length nor capitulum diameter was transgressive in any of the lines (Fig. 4). Eight of the families had style branch lengths outside the means of the parents. The more cryptic floral traits, such as ligule width and involucral bract length and width, varied from being transgressive in all lines to being intermediate in about half of the lines (Fig. 4). Only one of four fruit traits was transgressive in some lines and self-seed set in hybrids was intermediate between the parental species in every inbred line (Fig. 5).

**Figure 6.**
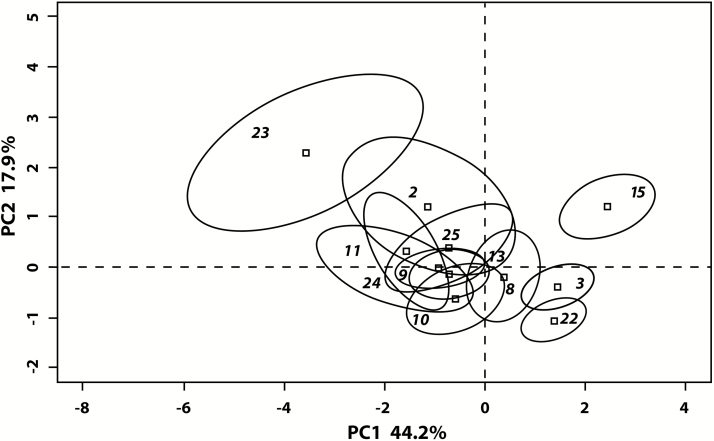
PCA of floral and vegetative characters with 95 % confidence ellipses around the barycentre of each inbred line. The first principal component (PC1) explains 44.2 % of the variance in the data set, and the second principal component (PC2) explains 17.9 %. A complex cluster of hybrid intermediacy is seen, but lines 3, 15, 22 and 23 are clearly distinct from others.

Results of the PCA of floral and vegetative characters are shown in Fig. 6. The 95 % barycentric confidence ellipses around group centroids show a large cluster of phenotypic overlap, but there is evidence of the formation of distinct phenotypes, e.g. lines 3, 15, 22 and 23 (Fig. 6). The first principal component has large eigenvectors for vegetative characters, which are relatively much smaller in magnitude on PC2 **[see Supporting Information—Table S1]**. On the other hand, PC1 has mostly negative eigenvectors for floral characters, which are positive on PC2. Only one floral trait, style branch length, loaded with vegetative characters, and no vegetative characters loaded with floral characters. Because principal components are uncorrelated by nature, it is clear that there exists little linkage between floral and vegetative characters in hybrid lines.

**Figure 7.**
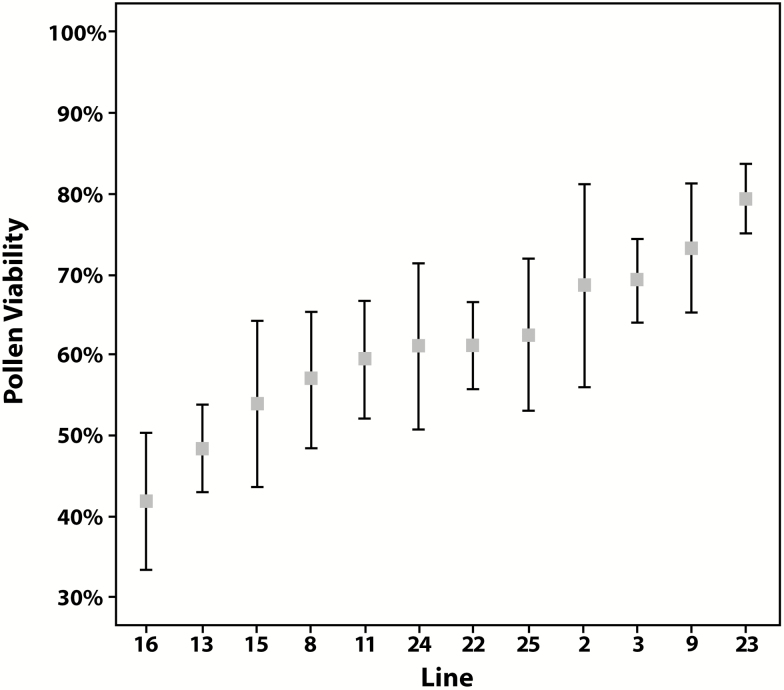
Plot of pollen viability in the F_4_ generation lines. Squares denote means and lines standard errors in each of the lines.

In all F_4_ generation lines, mean values for pollen viability were above 40 % (Fig. 7). However, there was variation in mean values among lines (42–79 %) and extensive variation among individuals within some lines (Fig. 7). Four of the lines were fixed for pappus presence, three lines had only plants lacking a pappus, and both conditions existed among individuals in the other lines.

**Figure 8.**
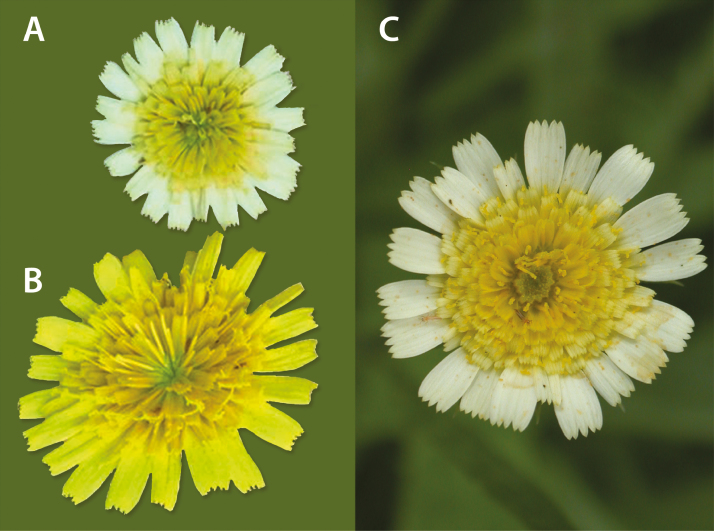
Capitula colour variation among several *Tolpis* lineages. (A) The rare, white floral form seen in some hybrid lines. Photo J. Ressler. (B) The typical yellow ligules found in most hybrid plants and all endemic Canarian species of *Tolpis*. Photo J. Ressler. (C) The capitulum of the non-endemic *Tolpis barbata*, grown from seed of *Crawford, Santos & Mort 1840A* collected near Santiago del Tiede, Tenerife. Photo J. K. Archibald.

A character that emerged in the hybrids that has not been detected in any other *Tolpis* endemic to the Canary Islands is white corollas in the outer florets of the capitula (Fig. 8A). This character was not fixed in any of the F_4_ inbred lines; rather, it was seen in four of the lines and only in line 13 was it present in more than one plant. The F_3_ maternal plant of line 13 represented the first appearance of this floral trait in any of the hybrid lines, and five of the 34 progeny (*ca.* 13 %) from this plant displayed white florets.

## Discussion

Our results demonstrate experimentally the generation of considerable variation in several characters within and across the 12 F_4_ inbred lines derived from a single cross between two species of *Tolpis*. For example, self-seed set varied within the F_4_ lines; this was more pronounced in some than in others (Fig. 5). An earlier study by Soto-Trejo *et al.* (2013) indicated that self-seed set is controlled by a major locus because there was a bimodal distribution of seed set in the F_2_ generation, although with a range of values in each of the groups. Even when Soto-Trejo *et al.* (2013) used a cut-off of 30 % or less seed set for SI and 50 % or more for SC, there still was a ratio of 2.94:1 for SI:SC (expected 3:1). Since seeds for succeeding generations originated from selfing, and there is evidence that self-seed set is controlled largely by a recessive allele at a single locus, one would expect the fixation for high self-seed set in each of the lines. The lower seed set may be a reflection of one or a combination of several factors discussed by Soto-Trejo *et al.* (2013), including the influence of modifier loci and pollen viability of parents.

In natural populations of Canary Island *Tolpis*, fruits without a pappus are rarely seen (Fig. 2B; Jarvis 1980; D. J. Crawford, University of Kansas, unpubl. data); however, plants lacking a pappus are unusually common in the population of the SI/PSC parent used in the initial cross in this study (10 %; D. J. Crawford, University of Kansas, unpubl. data). Three F_4_ lines appear to be fixed for pappus absence, and if verified with additional progeny, this would distinguish those lines from all other *Tolpis* endemic to the Macaronesian archipelagos. In addition to being an easily recognizable phenotypic trait (Fig. 2), the lack of a pappus in *Tolpis* diminishes the capacity for wind dispersal (K. J. Niklas *et al.*, Cornell University, unpubl. data).

There are few transgressive traits in *Tolpis* hybrid lines, especially for floral and seed characters. Stelkens and Seehausen (2009) concluded that genetic distance between parental species is positively correlated with transgressive traits in their hybrids, and that strong directional selection on traits is not conducive to the appearance of transgressive traits in the hybrids It is not known whether these two factors are responsible for the low frequency of transgressive traits in *Tolpis* hybrids. However, the lack of ITS sequence variation between the parental species (Mort *et al.* 2007) and likely strong selection for the selfing syndrome (Ornduff 1969; Foxe *et al.* 2009; Guo *et al.* 2009; Slotte *et al.* 2012; Soto-Trejo *et al.* 2013) suggest that the two factors are viable hypotheses for the relative lack of transgressive traits.

The most visibly striking transgressive character in the hybrids is the presence of white corollas on the outer florets of capitula (Fig. 8A) as opposed to the common condition of only yellow corollas (Fig. 8B). As far as we are aware, this colour variant has not been detected in any *Tolpis* endemic to the Canary Islands. However, a similar pattern of pigmentation is sometimes seen in *Tolpis barbata* (Fig. 8C), a species that occurs in, but is not endemic to the Macaronesian islands (Jarvis 1980). It is widely distributed in southern Europe and northern Africa (Jarvis 1980). Whether the similar colour patterns have the same genetic basis is not known.

Like floral traits, leaf characters in the hybrids are a mixture of intermediate and transgressive traits (Fig. 3), but all seed traits are intermediate between the parents (Fig. 5). In addition to transgressive traits, intermediate phenotypes can be viewed as novel in the sense that they do not occur in either of the parents. For example, García-Verdugo *et al.* (2013) pointed out that interspecific hybrids in Hawaiian *Dubautia* with leaf areas intermediate between the parental species occupy habitats distinct from each of the parents. They suggested that the variation in area and other leaf traits could facilitate the establishment and persistence of the hybrids in microenvironments where the parents were not seen.

In *Tolpis*, leaf perimeter (which reflects leaf dissection) was intermediate between the extremes of the parental species in nearly all hybrid lines (Figs 1B and 3). An array of leaf forms similar to those seen in the hybrids is known within *Tolpis* in the Canarian archipelago (Jarvis 1980; Crawford *et al.* 2009). *Tolpis* occurs in different vegetation zones (Bramwell and Bramwell 2001), but there are no studies of the correlation between leaf morphology and habitat.

Several of the inbred lines form morphologically distinct cohesive lineages (Fig. 6), and may be identified using a combination of characters. Whether or not any of these phenotypically distinct lines would be recognized as distinct species if they were found in nature is an open question, and ultimately a matter of judgment. Although there is no direct evidence that any of the synthetic hybrid lines would be reproductively isolated from each other, or from their parents in nature, there is reason to believe that gene flow could be reduced. All of the hybrid lines have relatively high levels of self-seed set and can self-pollinate, two attributes that would reduce gene flow among the lines (Levin 2002). Empirical and simulation studies indicate that selfing reduces gene flow via pollen, which would be effective in isolating hybrids from an outcrossing progenitor (Wright *et al.* 2013; Brys *et al.* 2014; Hu 2015). Although different inbred lines would initially be isolated primarily by mating system, other isolating barriers could subsequently evolve (Wright *et al.* 2013). The ability to self could drive the rapid evolution and fixation of characters (Foxe *et al.* 2009; Guo *et al.* 2009). Selfing could facilitate the initial establishment and persistence of small sexually reproducing founder hybrid populations because selfing could provide ‘reproductive assurance’ when compatible mates and pollinators are limited (Wright *et al.* 2013; Barrett *et al.* 2014; Barrett and Crowson 2016). In the heterogeneous landscape of the Canaries (Carracedo and Day 2002; Carracedo 2011), this may facilitate the colonization of open or disturbed areas not occupied by their parents (e.g. Brochmann *et al.* 2000; Francisco-Ortega *et al.* 2000; van Hengstum *et al.* 2012). The level of inbreeding depression in the selfing hybrids is not known and this could be a factor reducing fitness in the hybrids. For example, Layman *et al.* (2017) demonstrated that high seed discounting and inbreeding depression likely accounted for the maintenance of outcrossing despite the constant input of SC mutations.

Although one of the parents (*T. coronopifolia*) is an annual, all of the hybrids match the other parent (*T. santosii*) in being perennial. The combination of the perennial habit with the capacity for self-seed set would be an advantage in the establishment and persistence of new hybrid populations because individuals could persist even if there were low seed set or suboptimal conditions for seed germination in any given year, particularly in the early stages of population establishment.

## Conclusions

Twelve synthetic hybrid lines were generated from two species endemic to the Canary Islands. The parental species are closely related and genetically similar, but divergent in a number of phenotypic traits and in reproductive biology. The hybrid lines are pollen fertile, SC, perennials. Progeny from these lines exhibit combinations of characters not seen in either parent, have characters intermediate between their parents and display some transgressive traits relative to their parents. Morphometric analyses of floral and vegetative traits resolved several of the 12 lines as phenotypically distinct. The phenotypic novelty seen in the synthetic hybrids suggests evolutionary potential, including the possible origin of new homoploid hybrid species in heterogeneous landscapes such as those on the Canary Islands.

## Accession Numbers

Vouchers of the parental species used to generate F_4_ progeny are deposited in KANU under accession numbers: *Tolpis santosii* 397175 and *T. coronopifolia* 397158. High-quality digital images of the leaves of F_4_ progeny are available upon request.

## Supporting Information

The following additional information is available in the online version of this article—

**Table S1.** Loading of PCA analysis.

## Sources of Funding

This work was supported by a General Research Fund grant from the Department of Ecology and Evolutionary Biology at the University of Kansas to M.E.M.; J.R. and B.K. were supported by National Science Foundation Grant DBI-1262795 to J. Gleason and M.E.M.

## Contributions by the Authors

B.K., J.R. and M.J.S.B. collected and analysed the data; J.K.K. advised on data analyses; M.E.M. assisted in the collection of materials in the field, in the propagation of the experimental hybrids and facilitated funding for the research; A.S.-G. directed field work and the collection of materials; J.C.-C. facilitated field work and provided interpretations of the results; D.J.C. conceived of the project, made the crosses in the greenhouse, and was the primary writer, with extensive input from all other authors.

## Conflicts of Interest

None declared.

## Supplementary Material

Supporting_Information
