# Supplementary material for: The potential role of hybridization in diversification and speciation in an insular plant lineage: insights from synthetic interspecific hybrids
Source: AoB Plants. 2017 Sep 1;9(5):plx043. doi: 10.1093/aobpla/plx043 (PMC5714139; doi:10.1093/aobpla/plx043)
Supplement: Supporting_Information [file plx043_suppl_supporting_information.docx]

Table S1: Loadings of the PCA analysis

| Eigenvector | Correlation PC1 | P-value | Correlation PC2 | P-value |
| --- | --- | --- | --- | --- |
| LeafLength | 0.8997 | < 0.0001 | 0.2358786 | 1.23E-03 |
| LeafPerim | 0.8645 | < 0.0001 | 0.24054284 | 9.73E-04 |
| LeafWidth | 0.8643 | < 0.0001 | 0.15286709 | 3.78E-02 |
| LeafArea | 0.8328 | < 0.0001 | 0.08594321 | 2.45E-01 |
| StyleBranchLength | 0.1201 | 1.04E-01 | 0.46560275 | < 0.0001 |
| BractWidth | -0.0896 | 2.25E-01 | 0.09408316 | 2.03E-01 |
| LiguleWidth | -0.1956 | 7.62E-01 | 0.65314164 | < 0.0001 |
| BractLength | -0.2080 | 4.50E-03 | 0.59642608 | < 0.0001 |
| LiguleLength | -0.3676 | < 0.0001 | 0.73529697 | < 0.0001 |
| CapitulumDiameter | -0.5599 | < 0.0001 | 0.26663466 | 2.44E-04 |
